# Hybrid total hip arthroplasty 28 years after Pol Le Cœur triple pelvic osteotomy used for the treatment of developmental dysplasia of the hip: a case report

**DOI:** 10.1093/jscr/rjaf577

**Published:** 2025-09-21

**Authors:** Daniela M Isidoro, João Castro Mendes, Patrícia Dias, Catarina Corte-Real, Maria Alves Parreira, Diana Bicas Machado, Diogo Lino Moura, Fernando Judas

**Affiliations:** Department of Orthopedic Surgery and Traumatology, ULS Coimbra, Praceta Prof. Mota Pinto, 3004-561 Coimbra, Portugal; Department of Orthopedic Surgery and Traumatology, ULS Coimbra, Praceta Prof. Mota Pinto, 3004-561 Coimbra, Portugal; Department of Orthopedic Surgery and Traumatology, ULS Coimbra, Praceta Prof. Mota Pinto, 3004-561 Coimbra, Portugal; Department of Orthopedic Surgery and Traumatology, ULS Coimbra, Praceta Prof. Mota Pinto, 3004-561 Coimbra, Portugal; Department of Orthopedic Surgery and Traumatology, ULS Coimbra, Praceta Prof. Mota Pinto, 3004-561 Coimbra, Portugal; Department of Orthopedic Surgery and Traumatology, ULS Coimbra, Praceta Prof. Mota Pinto, 3004-561 Coimbra, Portugal; Department of Orthopedic Surgery and Traumatology, ULS Coimbra, Praceta Prof. Mota Pinto, 3004-561 Coimbra, Portugal; Department of Orthopedic Surgery and Traumatology, ULS Coimbra, Praceta Prof. Mota Pinto, 3004-561 Coimbra, Portugal; Faculdade de Medicina da Universidade de Coimbra, Polo III, Azinhaga de Santa Comba, 3000-548 Coimbra, Portugal

**Keywords:** developmental dysplasia of the hip, acetabular dysplasia, Pol Le Cœur triple pelvic osteotomy, total hip arthroplasty, hybrid total hip arthroplasty

## Abstract

A 35-year-old female patient with acetabular dysplasia underwent a Pol Le Cœur triple pelvic osteotomy (TPO). Twenty-eight years later, she presented with painful end-stage hip osteoarthritis and underwent total hip arthroplasty (THA). A press-fit cementless acetabular cup and a cemented straight femoral stem were implanted without complications. At 13-year follow-up, hip radiographs revealed no signs of prothesis instability and no signs of implant loosening. TPO may not completely prevent the progression of hip osteoarthritis. In this case, the patient developed progressive end-stage osteoarthritis 28 years after the osteotomy, requiring THA. Pol Le Cœur TPO is a viable treatment option for symptomatic acetabular dysplasia in younger adults with low-grade osteoarthritis, effectively delaying hip prosthesis implantation. Hybrid THA after TPO can yield very satisfactory clinical and radiographic outcomes at mid-term follow-up.

## Introduction

Surgical correction of idiopathic developmental dysplasia of the hip (DDH) is a challenging problem in orthopedic surgery, as untreated DDH may lead to the development of early arthritis [1, 2].

Triple pelvic osteotomy (TPO) is widely used to treat DDH in adolescents and young adults, especially those with unfused triradiate cartilage. The goal is to correct the acetabular position and achieve stable, congruent, and concentric coverage of the femoral head [1].

While pelvic osteotomy can improve joint function, it may not completely prevent the need for future surgeries. Some patients eventually require conversion to total hip arthroplasty (THA) for painful osteoarthritis.

THA is an accepted treatment for end-stage hip conditions, with excellent long-term outcomes and high success rate [2]. Some studies suggest that THA after previous pelvic osteotomy presents technical challenges and may yield inferior outcomes compared to primary THA, while others report contradictory results. The acetabulum may present deformation and may also exhibit posterior wall deficiency [3].

We presented a case of an adult female patient who underwent THA 28 years after a Pol Le Cœur TPO, a procedure performed to manage low-grade acetabular dysplasia.

## Case report

**Figure 1:**
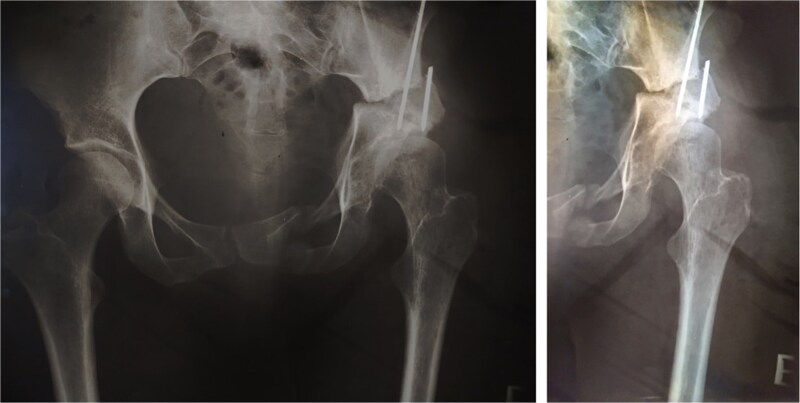
Postoperative anteroposterior radiographs of the pelvis, showing Pol Le Cœeur triple pelvic osteotomy performed on a 35-year-old female patient to treat an acetabular dysplasia in the left hip, at Grade 1 Crowe’s classification.

In 1984, a 35-year-old female patient presented to our department with severe left groin pain. Preoperative pelvic radiographs revealed acetabular dysplasia with subluxation at Crowe Grade I and Tönnis Grade I. A Pol Le Cœur corrective TPO was performed (Fig. 1). Over the following 28 years, radiographic examinations demonstrated progressive articular degradation despite the initial clinical success of the osteotomy.

**Figure 2:**
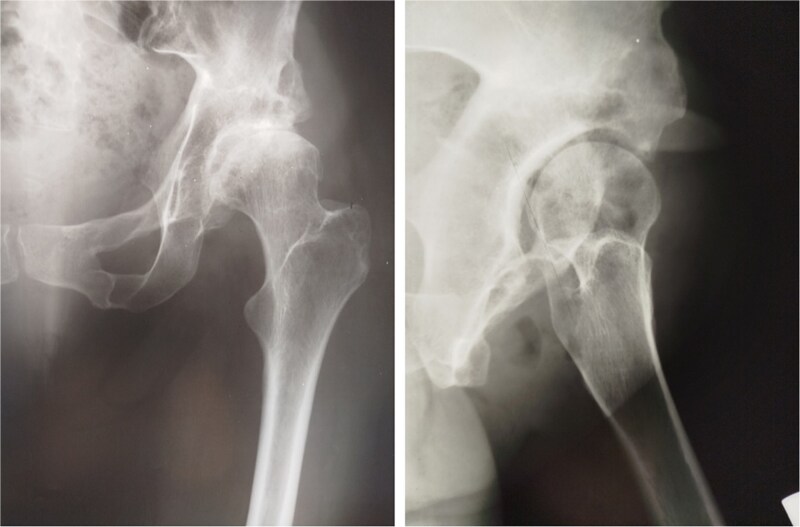
Postoperative radiographs of the pelvis 20 years after pelvic osteotomy, showing signs of moderate osteoarthritis in the left hip (Tönnis Grade III), and radiographic evidence of acetabular retroversion (ischial spine sign and posterior wall sign).

At 20-year follow-up, she presented with symptomatic hip pain. Pelvic radiographs (Fig. 2) showed signs of moderate osteoarthritis in the left hip (Tönnis Grade III). Radiographs showed signs of acetabular retroversion (ischial spine sign and posterior wall sign).

**Figure 3:**
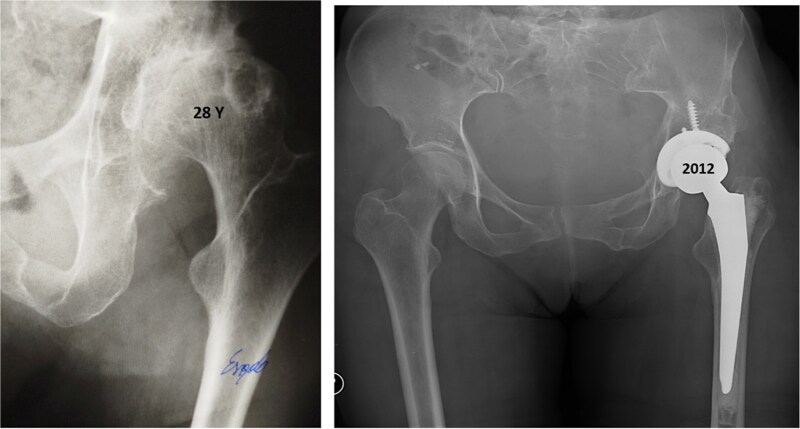
Anteroposterior radiographs of the pelvis 28 years after the pelvic osteotomy, showing advanced left hip osteoarthritis (Tönnis Grade V). A hybrid cemented total hip arthroplasty was implanted using a standard posterolateral approach.

Twenty-eight years after triple osteotomy, the patient presented with painful end-stage osteoarthritis, with body mass index of 18. A hybrid cemented THA was performed using a standard posterolateral approach (Fig. 3). A cobalt-chrome femoral head was articulated with highly reticulated polyethylene liner. A press-fit cementless cup stabilized with two screws (Tylogy®, Zimmer) and a cemented straight femoral stem (Müller®, Zimmer) were implanted.

The postoperative course was uneventful, and the patient quickly resumed daily activities. No relevant comorbidities were recorded.

**Figure 4:**
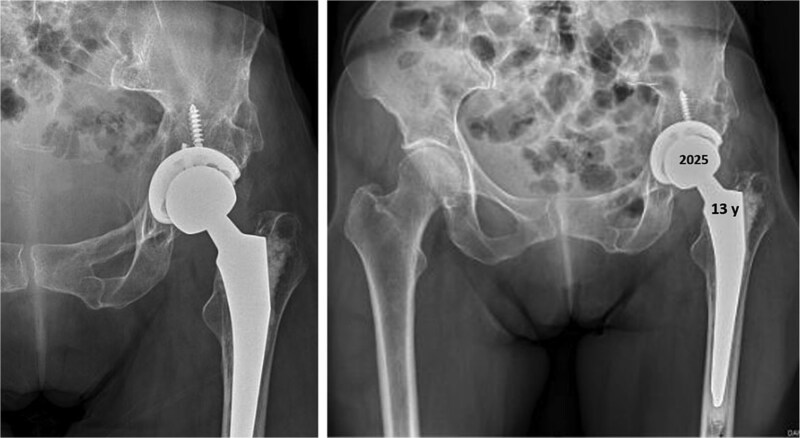
Postoperative anteroposterior radiographs of the pelvis 13 years after the THA, showing mechanical stability of the prosthesis with no evidence of loosening. No signs of osteoarthritis are present in the right hip (Tönnis Grade 0).

Thirteen years after THA, the 76-year-old patient continued to have a stable asymptomatic hip. Hip radiographs showed unchanged and stable positioning of the acetabular cup and femoral stem, with no measurable subsidence or radiolucent lines around the components (Fig. 4).

## Discussion

In 1984, the 35-year-old patient underwent a Pol Le Cœur triple pelvic osteotomy for the management of Crowe Type I acetabular dysplasia. Joint-preserving pelvic osteotomies are an established treatment for adult hip pain secondary to DDH [4].

Pelvic osteotomies are grouped into reorientation (periacetabular, triple), redirectional (Salter, Pemberton, Dega), and salvage (Chiari) types [5]. Triple osteotomy shows satisfactory results and remains a viable option [6].

In this case, a corrective Pol Le Cœur osteotomy was employed for the management of the acetabular dysplasia, showing a satisfactory long-term clinical result. The concept of rotating the acetabulum over the femoral head developed into TPO was first described by Pol Le Cœur in 1965 [7]. The goal is to achieve sufficiently extensive contact between the femoral and acetabular articular cartilages. To accomplish this, the Le Cœur osteotomy osteotomizes the pubic and ischial rami closer to the pubic symphysis compared to the steel procedure [8]. The tilting of the acetabulum on the femoral head must be adequate, meaning technically feasible, and the amount of existing cartilage must be sufficient [7, 9].

Despite TPO can significantly improve hip function and reduce pain, it does not eliminate the risk of developing a progressive painful osteoarthritis. In 2012, 28 years after pelvic osteotomy, the patient underwent THA due to a symptomatic end-stage left hip osteoarthritis, with articular collapse at Tönnis V stage.

Pain and severe functional impairment resulting from end-stage osteoarthritis of the hip after a pelvic osteotomy are a clear indication for THA [10]. THA following previous pelvic osteotomy is associated with increased intraoperative time and blood loss, as well as greater acetabular anteversion angle and larger cup size, and a more proximal and lateral joint center [3]. Placement of acetabular component plays a critical role in instability, range of motion, bearing surface wear rates, and survivorship [11].

Inappropriate acetabular component positioning can lead to dislocation, component impingement, dysmetria, and mechanical failure. Acetabular retroversion and insufficient posteroinferior wall could affect cup alignment, mechanical stability, and the type of acetabular reconstruction [10, 12]. The use of preoperative computed tomography scan facilitated the evaluation of the three-dimensional acetabular construction [3, 10], and robotic-assisted THA may simplify these complex procedures [11].

THA following previous pelvic and femoral osteotomy provides pain relief and improved function with similar complication rates, clinical outcomes, and survivorship compared to hips undergoing routine primary THA [5, 13].

In this case, a hybrid hip prosthesis, consisting of a cementless primary cup and a cemented straight femoral stem, was implanted using a standard posterolateral approach. On the acetabular side cemented and cementless components, press-fit cups with or without screw fixation, metal augments, metal rings and cages, and bone grafts can be used. Favorable results were described using metal rings in complex primary or revision hip arthroplasty [14].

Due to femoral proximal deformities and excessive anteversion of the femur in hip dysplasia, a cementless femoral stem with conical shape, and with a rounded cross-section can be indicated. The conical fixation of the stem ensures excellent primary stability. It is possible to produce correct femoral anteversion with no technical difficulties [15].

Thirteen years after the arthroplasty surgery, the 76-year-old patient presented an asymptomatic hip. The hip radiographs showed a satisfactory orientation of the prosthesis and no signs of mechanical instability or radiolucent lines around the prosthesis.

Patient-related factors, such as low body mass index and limited physical demands, may have contributed to the favorable clinical outcome.

This case demonstrates that hybrid THA after pelvic osteotomy can result in favorable mid-term outcomes when carefully planned.
